# Personal

**Published:** 1918-03

**Authors:** 


### PERSONAL

**Announcement of removal, travel, and other matters of interest are requested. Please report errors in the listing of any physician in the State and other directories, that we may co-operate with the State Society in securing a correct list.**

Dr. Harry C. Dumville of Niagara Falls has been elected President of the Managers of the Niagara Co. Tuberculosis Hospital at Lockport.

Dr. S. Tracey Hamilton, U. B. 1916, has opened offices at 52 Lake St., Elmira.

Dr. Charles Haase has been appointed Health Commissioner at Elmira.

Chemung County's Tuberculosis Hospital has been completed.

Mr. Joseph L. Turner, Head Chemist of the Bristol-Myers Co., Brooklyn, has been elected Vice-President of the New York Local Branch of the American Pharmaceutical Association.

Dr. Alfred H. Noehren announces the removal of his office and residence to 1196 Main St.

Dr. Monroe Manges is spending March in Florida.

Dr. E. W. Ewell of Lancaster, has been appointed town physician.

Dr. Renwick R. Ross, Supt. of the Buffalo General Hospital, will return this month from a two-months' trip to California.

The following appointments have been made, received just too late for insertion in the Feb. issue: Dr. George S. Staniland, member of the board of managers of city hospitals and dispensaries; Dr. Jesse N. Roe, diagnostician, temporarily, in place of Dr. Harold J. MacDonald who has entered military service.

The Dunkirk Board of Health has made the following reelections: Dr. George E. Ellis, City Health Officer, Dr. C. E. Hallenbeck, Bacteriologist.

Dr. R. B. Wakeman of Hornell has been appointed president of the board of managers of the Steuben Co. Tuberculosis Hospital and Dr. E. E. Webster of Woodhull is also a member of the board.

Dr. Edward Clark has resumed his former duties as sanitary supervisor for the 19th district which comprises Buffalo and Western New York counties. Dr. H. F. Sinftner will be the acting director of the division of child hygiene.

Dr. Daniel Jong, Buffalo General Hospital, Dr. Frederick W. Palmer, 100 High St., and Dr. Antonio L. Baron, 246 Front Ave., have been commissioned first lieutenants in the Medical Reserve Corps.

The Annual Dinner of the Buffalo Health Bureau was held at the Hotel Iroquois Feb. 7. Health Commissioner Fronczak presided and the speakers were Mayor Buck and Dr. Albert H. Briggs, who was head of the department 38 years ago.

Dr. Fronczak said that in the past fifteen years the infant mortality rate has been reduced from 214 to 108 per 1,000 while the death rate in the past 25 years has been reduced from 23.9 to 14.21. Nine employees in the department are in military service.

